# Seafood consumption patterns and methylmercury risk awareness among Saudi adults: a nationwide cross-sectional survey documenting a structural knowledge–behavior gap

**DOI:** 10.3389/fpubh.2026.1886816

**Published:** 2026-07-20

**Authors:** Nawaf W. Alruwaili, Abdulaziz Mashraqi, Nora Alafif

**Affiliations:** 1 Department of Community Health Sciences, College of Applied Medical Sciences, King Saud University, Riyadh, Saudi Arabia; 2 Makkah Health Cluster, Makkah, Saudi Arabia

**Keywords:** cross-sectional survey, food safety communication, knowledge–behavior gap, methylmercury, risk awareness, Saudi Arabia, seafood consumption

## Abstract

**Background:**

Seafood provides essential nutritional benefits but represents the primary dietary route of exposure to methylmercury (MeHg). Population-level evidence linking adult seafood consumption behavior to MeHg risk awareness in Saudi Arabia remains absent. This study aimed to characterize seafood consumption patterns, estimate MeHg hazard recognition, and identify independent predictors of high-risk consumption and mercury risk awareness among Saudi adults.

**Methods:**

This nationwide cross-sectional survey enrolled 1,021 adults across all 13 Saudi administrative regions (January–April 2026) and used two pre-specified binary logistic regression models—one examining predictors of high-risk seafood consumption (Model 1) and one examining predictors of mercury risk awareness (Model 2).

**Results:**

Regular seafood consumption was reported by 47.4% of participants; only 19.7% identified mercury as a health concern. High-risk consumption, operationalized using FDA/U.S. EPA advisory thresholds and Gulf-region contamination data, was identified in 53.3% of participants (sensitivity range across five alternative definitions: 24.9–70.4%). Women demonstrated higher MeHg-specific awareness (23.6% vs. 16.5%; *p* = 0.005) and greater food-safety knowledge (1.29 ± 1.91 vs. 0.71 ± 1.42; *p* < 0.001). Food-safety knowledge strongly predicted mercury awareness (OR = 4.000, 95% CI [3.350–4.777]) but was not associated with consumption behavior (OR = 0.997; *p* = 0.950). Coastal residence (OR = 3.019), older age (≥50 years: OR = 3.494), female sex (OR = 0.534), and lower educational attainment independently predicted high-risk consumption.

**Conclusion:**

Consistent with our objective of evaluating whether food-safety knowledge translates into safer seafood choices, this knowledge–behavior gap was robust across all five sensitivity specifications, confirming a structural—rather than informational—dissociation between awareness and behavior. Species-specific, geographically targeted interventions, rather than general knowledge-based campaigns, are therefore required to reduce the risk of MeHg exposure among Saudi adults.

## 1 Introduction

Seafood occupies a well-established position in evidence-based dietary guidance, supplying high-quality protein, iodine, selenium, vitamin D, and the long-chain omega-3 polyunsaturated fatty acids—eicosapentaenoic acid (EPA) and docosahexaenoic acid (DHA)—whose contributions to cardiovascular health and neurodevelopmental outcomes across the life course are extensively documented (1–4). On the basis of this evidence, international nutritional and regulatory bodies have incorporated regular seafood intake into dietary recommendations for the general population (5–7). Those recommendations, however, cannot be considered independently of the principal contaminant risk posed by the same aquatic food supply: dietary exposure to methylmercury (MeHg), the foremost toxicological hazard associated with fish consumption (8–10).

In aquatic ecosystems, inorganic mercury is methylated by micro-organisms and subsequently concentrated through trophic biomagnification, yielding the highest MeHg burdens in large, long-lived, upper-trophic predatory fish (8, 11, 12). The toxicological significance of this pathway is amplified by the near-complete gastrointestinal absorption of methylmercury, its extended biological half-life, and its capacity to penetrate both the placental and blood–brain barriers, placing the developing nervous system at disproportionate risk (13, 14). Longitudinal cohort research, including the Seychelles Child Development Study (15–21), consistently associates prenatal MeHg exposure with neurodevelopmental consequences, although the magnitude of observed effects varies across cohort characteristics, exposure distributions, and analytical strategies (22–25). Chronic adult dietary exposure to high-MeHg predatory species can produce bioaccumulation sufficient to trigger sensory disturbances, cognitive deficits, motor incoordination, and cardiovascular harm (7, 26). International risk–benefit evaluations consistently affirm that the nutritional benefits of seafood outweigh MeHg-associated harms provided that lower-mercury species are preferentially consumed (5, 6, 27), prompting a shift toward species-specific advisory frameworks calibrated to mercury burden, consumer life stage, and product category (7, 28–30).

Notwithstanding this policy evolution, consumer awareness of fish advisories is frequently superficial, and even where awareness exists, it does not reliably translate into safer dietary choices (31–36). Seafood selection is shaped by a complex interplay of sensory preference, habit, affordability, cultural norms, and trust in food-safety institutions, rather than by toxicological information alone (37–42). This persistent disconnect between knowledge and consumption behavior—the knowledge–behavior gap—cross-cuts diverse regulatory systems and cultural contexts (43–46).

Saudi Arabia presents an underexplored national context in which these pressures converge. The country’s dual coastlines on the Red Sea and Arabian Gulf, together with an expanding fisheries and aquaculture sector under the Vision 2030 economic diversification agenda, have generated growing demand for and access to seafood (3, 47). The Saudi Ministry of Health has issued guidance on fish consumption for the general public (48), yet the extent to which this guidance reaches and influences dietary behavior remains unknown. Environmental monitoring studies from Saudi Arabia and the broader Gulf region have repeatedly documented elevated mercury concentrations in commercially important predatory fish from regional waters (49–55), and regional exposure studies confirm that MeHg risk varies substantially according to species selection and consumption frequency (56–58). Biomonitoring data in Saudi women further underscore the public health relevance of dietary exposure surveillance in vulnerable subgroups (59–62).

Despite this evidence, population-level Saudi data linking adult seafood consumption to MeHg-related knowledge and risk awareness—the knowledge, attitudes, and practices (KAP) dimension—have been almost entirely absent from the published literature. We hypothesized that objective food-safety knowledge would be positively associated with both higher mercury risk awareness and lower high-risk seafood consumption after adjustment for relevant sociodemographic covariates. Accordingly, this study was undertaken with three objectives: (1) to characterize seafood consumption patterns, purchasing determinants, and species preferences among adults across Saudi Arabia; (2) to assess the prevalence of food-safety knowledge and MeHg-specific risk awareness; and (3) to identify the independent sociodemographic predictors of high-risk seafood consumption behavior and mercury risk awareness.

## 2 Methods

### 2.1 Study design and ethical approval

A descriptive cross-sectional survey design was adopted. This approach permitted concurrent measurement of dietary behavior, food-safety knowledge, and risk awareness within a single data-collection window across a geographically dispersed adult population, an appropriate choice given the descriptive and exploratory objectives of this study. Ethical approval was obtained from the Subcommittee on Human and Social Research Ethics at King Saud University (Reference No. KSU-HE-26-0045) prior to data collection. All procedures conformed to the Declaration of Helsinki. Electronic informed consent was obtained from each participant before survey initiation; documentation addressed study objectives, voluntary participation, the unconditional right to withdraw, and assurances of data confidentiality and anonymity. Participant data were stored in encrypted, password-protected files accessible exclusively to the research team and will be retained for 7 years in accordance with institutional policy.

### 2.2 Study setting and participants

The study was carried out in the Kingdom of Saudi Arabia, encompassing all 13 administrative regions and capturing both coastal cities (Red Sea and Arabian Gulf littoral) and inland areas. The target population comprised adults aged 20 years and older residing in Saudi Arabia. This lower age threshold reflects the minimum age of independent food purchasing, aligns with the adult classification used in the source instrument (38) and prior Saudi dietary surveys, and corresponds to the population for whom public health risk communication on MeHg is most policy-relevant. Recruitment used a non-probability convenience sampling approach: a bilingual (Arabic/English) Google Forms questionnaire was distributed via social-media channels between January and April 2026. Participation was entirely self-selected: prospective participants opted in voluntarily by accessing the distributed survey link, with no researcher-directed selection or targeting of specific individuals. Respondents were drawn from all 13 administrative regions; however, this regional coverage reflects voluntary participation rates rather than population-proportional sampling and may not reflect each region’s population size. Survey responses were automatically filtered to exclude incomplete submissions (operationally defined as form sessions in which one or more mandatory items were not answered before the session was terminated; the Google Forms platform was configured to prevent submission unless all required fields were completed, ensuring that every retained response is complete on all mandatory items); 1,021 complete, valid responses were retained for analysis (estimated responses received: ~1,078; incomplete/duplicate exclusions: ~57; exact response tracking was precluded by the open-channel distribution method). Participant nationality was not collected.

### 2.3 Sample size and statistical power

A minimum sample size of 384 participants was estimated using Cochran’s formula (Z = 1.96; *p* = 0.50; d = 0.05; 95% confidence level). The target was raised to 425 to accommodate anticipated non-response; 1,021 valid responses were ultimately obtained. *Post-hoc* evaluation of statistical adequacy for the logistic regression analyses employed the events-per-variable (EPV) criterion (63): EPV = 68 for the high-risk consumption model (outcome prevalence 53.3%; 8 predictors) and EPV = 25 for the mercury risk awareness model (outcome prevalence 19.7%; 8 predictors), both substantially exceeding the recommended minimum of 10 EPV.

### 2.4 Survey instrument and cultural adaptation

The questionnaire was adapted from the validated instrument of Spagnolo et al. (38), developed to assess fish consumption patterns and health risk perception in an adult coastal Italian population. Cultural adaptation involved substituting the original species list with taxa prevalent in Saudi and Gulf markets, modifying market-source examples to reflect the local food retail landscape, and adjusting selected risk-awareness items to align with the Saudi food safety context, while preserving the conceptual and structural framework of the source instrument.

Translation followed a rigorous four-stage forward–backward procedure. Two postgraduate-level Arabic–English translators each produced independent forward translations from the English source. A third bilingual expert reviewed these translations and produced a reconciled Arabic consensus text. A fourth translator, deliberately kept blind to the English original, back-translated the consensus into English; the research team then compared the result with the source text to resolve any remaining conceptual divergences. A pilot run with 30 adults drawn from the target population (subsequently excluded from analysis) assessed item clarity and cultural fit; five questions were reworded before full deployment. The full bilingual (Arabic/English) survey instrument is provided as Supplementary File S3.

### 2.5 Variable operationalization

#### 2.5.1 High-risk seafood consumption

A binary outcome variable was operationalized according to species-specific MeHg burden criteria documented in international advisory frameworks and Gulf-region contamination literature. A participant was classified as a high-risk consumer if either of the following criteria applied: (1) any reported consumption of *Scomberomorus commerson* (Al-Kanaad), irrespective of frequency—this species is designated “Choices to Avoid” (mean mercury concentration ≥0.46 ppm) by the FDA/U.S. EPA (7), and Gulf monitoring studies report concentrations of 0.45–1.20 ppm from regional waters (51, 54); or (2) weekly-or-more-frequent consumption of *Epinephelus coioides* (Al-Hamour; 0.19–0.55 ppm), *Plectropomus pessuliferus* (Al-Najil; 0.20–0.45 ppm), or *Lethrinus nebulosus* (Al-Shaour; 0.10–0.35 ppm) (49, 55), corresponding to FDA/U. S. EPA “Good Choices” with a recommended ceiling of one serving per week. Thunnus spp. (tuna) was excluded as a standalone criterion because its mercury burden varies markedly by species and product form; canned light tuna from Gulf markets averages 0.09–0.28 ppm (64), within FDA/U.S. EPA Best or Good Choice thresholds. The robustness of this operationalization was evaluated across five alternative definitions in sensitivity analyses (Supplementary Table S2).

#### 2.5.2 Food-safety knowledge score

Participants were presented with a list of health hazards and asked to identify any they associated with seafood consumption. Six items were scored dichotomously (0 = not selected; 1 = selected): K1, general health risk recognition; K2, recognition of mercury or heavy metals as a seafood health hazard; K3, microorganisms; K4, veterinary drug or aquaculture residues; K5, preservatives or storage compounds; and K6, raw or undercooked fish. The composite Knowledge Total score (range 0–6) demonstrated satisfactory internal consistency (Cronbach’s *α* = 0.865; equivalent to Kuder–Richardson Formula 20 for binary items). A modified score, Knowledge′ (K1 + K3 + K4 + K5 + K6; range 0–5; α = 0.836), was used as the sole knowledge predictor in the mercury risk awareness regression model to preclude circular dependency between the predictor and the binary outcome (K2). The Knowledge distribution was severely right-skewed: mean = 0.78 (SD = 1.38), median = 0, and 71.6% of participants scored zero.

#### 2.5.3 Mercury risk awareness

A single binary variable derived from K2 captured whether the participant identified mercury or heavy metals as a health concern associated with seafood (0 = No; 1 = Yes). This item served as the outcome variable for the risk awareness regression model. Its single-item operationalization captures hazard recognition but does not encompass the multidimensional construct of risk perception; this limitation is acknowledged in the limitations and strengths section.

### 2.6 Statistical analysis

All analyses were performed using IBM SPSS Statistics Version 30.0 (65). Two binary logistic regression models, estimated using the Enter (simultaneous/forced-entry) method, were used to identify independent predictors of (1) high-risk seafood consumption (Model 1) and (2) mercury risk awareness (Model 2). Categorical variables are presented as frequencies and percentages; for multi-response items, percentages were computed relative to respondents who answered each item and may therefore exceed 100%. Continuous variables are reported as means and standard deviations. Bivariate associations were assessed with Pearson’s chi-square (without continuity correction) and independent-samples t-tests, as appropriate. For Model 1, candidate predictors included age group, sex, educational level, coastal residence, and Knowledge Total; all five predictors were retained in the final model. For Model 2, all five predictors (Knowledge, sex, age group, educational level, and coastal residence) were retained. Multicollinearity was assessed via variance inflation factors (all VIF ≤ 1.61). Model fit was evaluated using the omnibus chi-square, Nagelkerke R^2^, area under the receiver-operating characteristic curve (AUC), and the Hosmer–Lemeshow (H–L) statistic; because the H–L test has well-documented inflated Type I error in large samples (n ≥ 500), AUC was treated as the primary calibration index for Model 2 (66). Calibration was additionally assessed using the Spiegelhalter Z-statistic (67), selected because it remains valid for binary logistic regression under large-sample distributional conditions in which the Hosmer–Lemeshow test over-rejects. Results are expressed as odds ratios (ORs) with 95% confidence intervals (CIs); two-tailed *p* < 0.05 was the significance threshold. Nonparametric verification using the Mann–Whitney U test confirmed identical conclusions across all t-test comparisons (*p* < 0.001). The prevalence of high-risk consumption was additionally evaluated across five alternative outcome specifications in sensitivity analyses. This study was reported in accordance with the STROBE statement; a completed STROBE checklist is provided as Supplementary Table S1.

## 3 Results

### 3.1 Sociodemographic characteristics

Table 1 presents the characteristics of the 1,021 participants. Men constituted a slight majority (55.1%), and the cohort was relatively young, with adults aged 20–39 years accounting for more than 70% of participants. Geographically, 45.8% resided in coastal cities and 54.2% in inland areas. The sample was highly educated: 57.4% held a bachelor’s degree and 17.1% postgraduate qualifications. Approximately half were married (50.5%), 76.4% lived in households of four or more people, 78.8% reported having children at home, and 52.9% identified as the primary grocery shopper.

**Table 1:** Sociodemographic characteristics of the study population (*N* = 1,021).

Variable	Category	*n* (%)
Sex	Male	563 (55.1)
	Female	458 (44.9)
Age (years)	20–29	463 (45.3)
	30–39	261 (25.6)
	40–49	205 (20.1)
	≥50	92 (9.0)
Marital status	Single	505 (49.5)
	Married	516 (50.5)
Coastal residence	Yes	468 (45.8)
	No	553 (54.2)
Educational level	Below bachelor’s degree	260 (25.5)
	Bachelor’s degree	586 (57.4)
	Postgraduate	175 (17.1)
Household size	<4 members	241 (23.6)
	≥4 members	780 (76.4)
Children in household	Yes	805 (78.8)
	No	216 (21.2)

Postgraduate includes Higher Diploma, Master’s, and Doctoral qualifications.

### 3.2 Seafood consumption and purchasing determinants

Almost half of the adults surveyed (47.4%) identified themselves as regular seafood consumers. Purchasing decisions were driven primarily by quality and freshness (71.0%), followed by price (51.4%) and health or origin considerations (45.7%). Consumption frequency by product type is presented in Table 2. Fresh seafood showed the most varied distribution, with 38.5% consuming it weekly or more frequently; canned seafood showed high regularity (47.2% weekly or more); frozen seafood was consumed less frequently, with 29.1% never consuming it. The modal portion size was ≤100 g (34.3%; Table 3). Buyers strongly preferred traditional local markets (75.6%; Table 4), with supermarkets serving as a secondary source (42.1%).

**Table 2:** Seafood consumption frequency by product type (*N* = 1,021).

Frequency	Fresh *n* (%)	Frozen *n* (%)	Canned *n* (%)
Never	69 (6.8)	297 (29.1)	86 (8.4)
Rarely (<monthly)	212 (20.8)	217 (21.3)	119 (11.7)
Monthly	159 (15.6)	160 (15.7)	137 (13.4)
Bi-weekly	188 (18.4)	162 (15.9)	197 (19.3)
Weekly	169 (16.6)	96 (9.4)	186 (18.2)
Several times/week	124 (12.1)	43 (4.2)	169 (16.6)
Daily	100 (9.8)	46 (4.5)	127 (12.4)

Rows sum to 100% within each product type.

**Table 3:** Distribution of typical seafood portion sizes (*N* = 1,021).

Portion size	*n* (%)
≤100 g	350 (34.3)
101–125 g	179 (17.5)
126–150 g	272 (26.6)
151–175 g	101 (9.9)
176–212.5 g	71 (7.0)
≥250 g	48 (4.7)

Data represent self-reported typical portion sizes per eating occasion.

**Table 4:** Place of purchase for seafood products.

Place of purchase	*n*	%
Local fish market	772	75.6
Supermarket/hypermarket	430	42.1
Discount store	45	4.4
Online platform	38	3.7

Percentages exceed 100% because respondents could select multiple options.

Barriers to higher consumption are detailed in Table 5. A general lack of dietary habits constituted the primary barrier (45.1%), followed by high price (38.9%) and preparation challenges (27.6%). Notably, health and safety concerns were cited by only 7.0% of respondents, affirming the low salience of contaminant risk at the point of food decision. Species preferences are reported in Table 6. Tuna (54.9%) and shrimp (48.5%) were most frequently consumed. A substantial proportion also reported regular consumption of high-trophic predatory species: 44.9% consumed greasy grouper (*Epinephelus coioides*; Al-Hamour) and 44.6% consumed narrow-barred Spanish mackerel (*Scomberomorus commerson*; Al-Kanaad), both of which carry elevated mercury burdens in Gulf waters (51, 52, 55).

**Table 5:** Reported reasons for low seafood consumption.

Reason	*n*	%
Lack of dietary habit/routine	435	45.1
High price	375	38.9
Preparation challenges	266	27.6
Taste preference	192	19.9
Health and safety concerns	67	7.0

Percentages exceed 100% because respondents could select multiple options; denominator = all 1,021 participants.

**Table 6:** Seafood species commonly consumed by the study population.

Seafood type	*n* (%)
Tuna (*Thunnus* spp.)	553 (54.9)
Shrimp (*Penaeus* spp.)	488 (48.5)
Greasy grouper (*Epinephelus coioides*; Al-Hamour) †	452 (44.9)
Narrow-barred Spanish mackerel (*Scomberomorus commerson*; Al-Kanaad) †	449 (44.6)
Atlantic salmon (*Salmo salar*) ‡	328 (32.6)
Spangled emperor (*Lethrinus nebulosus*; Al-Shaour) †	301 (29.9)
Leopard coral grouper (*Plectropomus pessuliferus*; Al-Najil) †	145 (14.4)
European seabass (*Dicentrarchus labrax*) ‡	98 (9.7)
White-spotted spinefoot (*Siganus canaliculatus*)	38 (3.8)

Percentages do not sum to 100% because participants could select multiple species. † Species with documented elevated mercury burdens in Arabian Gulf/Red Sea waters (49–52, 55). ‡ Atlantic salmon and European seabass are primarily farmed or imported species with generally low mercury content; not included in the high-risk classification.

### 3.3 Sex differences in risk perception and knowledge

High-risk seafood consumption was identified in 53.3% of participants (*n* = 544). Overall, mercury-specific awareness was low: 19.7% (*n* = 201) identified mercury as a health concern. Women demonstrated significantly higher awareness than men (23.6% vs. 16.5%; χ^2^ = 7.966, *p* = 0.005; Cramér’s V = 0.09) and higher Knowledge Total scores (1.29 ± 1.91 vs. 0.71 ± 1.42; *t* = −5.619, *p* < 0.001; Cohen’s d = 0.35; Table 7). Mann–Whitney U confirmed both findings (*p* < 0.001). Conversely, men showed substantially higher rates of high-risk consumption (65.9% vs. 37.8%; χ^2^ = 80.25, *p* < 0.001; V = 0.28), the largest bivariate effect size observed in the dataset.

**Table 7:** Sex differences in mercury risk awareness, food-safety knowledge, and high-risk consumption (N = 1,021).

Variable	Male (*n* = 563)	Female (*n* = 458)	Statistic	*p*-value	Effect size
Mercury awareness, *n* (%)	93 (16.5)	108 (23.6)	χ^2^ = 7.966	0.005	V = 0.09
Knowledge total, Mean ± SD	0.71 ± 1.42	1.29 ± 1.91	t = −5.619	<0.001	d = 0.35
High-risk consumption, *n* (%)	371 (65.9)	173 (37.8)	χ^2^ = 80.25	<0.001	V = 0.28

MeHg, methylmercury; SD, standard deviation; V = Cramér’s V; d = Cohen’s d. Cramér’s V benchmarks: small <0.10, medium 0.10–0.30. Cohen’s d benchmarks: small ≈0.20, medium ≈0.50. Chi-square without continuity correction. Knowledge total: Cronbach’s α = 0.865; range 0–6. Mann–Whitney U confirmed all *t*-test conclusions (*p* < 0.001).

### 3.4 Knowledge score distribution

**Figure 1:**
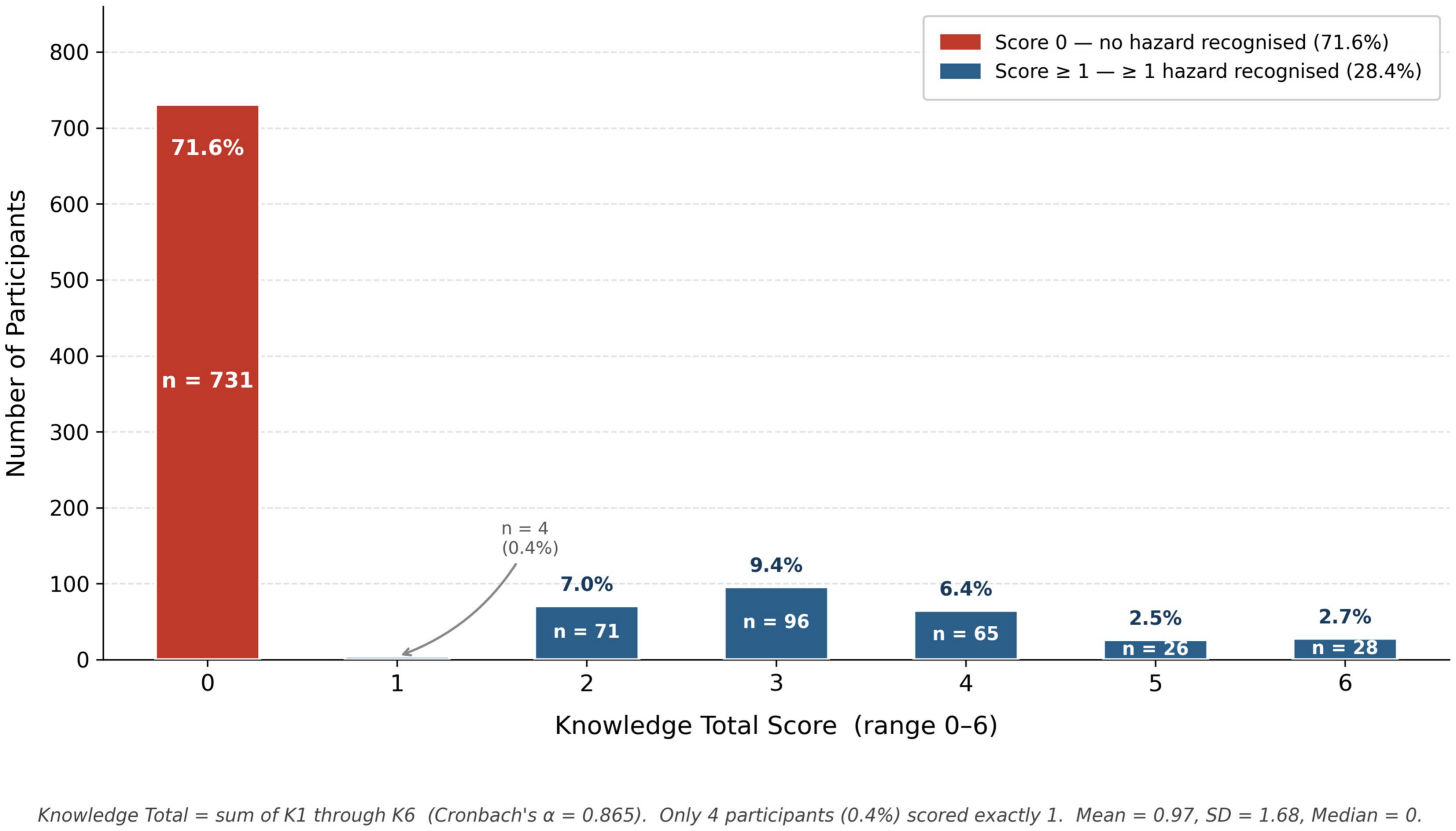
Distribution of knowledge total scores (*N* = 1,021). The composite (K1 + K2 + K3 + K4 + K5 + K6; range 0–6; Cronbach’s *α* = 0.865) displays severe zero-inflation: 71.6% of participants (n = 731) scored zero, indicating no recognition of any listed seafood health hazard. Only 4 participants (0.4%) scored exactly 1. Mean = 0.97, SD = 1.68, Median = 0. The near-absence of score 1 reflects a pattern in which participants who recognized any hazard tended to recognize multiple hazards simultaneously.

The Knowledge Total composite showed severe zero inflation. Of 1,021 participants, 71.6% (*n* = 731) scored zero, indicating no recognition of any listed seafood health hazard. Mean score was 0.97 ± 1.68 (range 0–6); median = 0. Only 4 participants scored exactly 1, suggesting that participants who recognized any food-safety hazard typically recognized multiple hazards simultaneously—consistent with food-safety literacy being acquired as a gestalt rather than item-by-item. The modified Knowledge′ composite (K1 + K3 + K4 + K5 + K6; K2 excluded) showed an identical zero-inflation pattern (71.6% scoring zero; mean = 0.78 ± 1.38). This distributional structure is central to the interpretation of Model 2 (Section 3.6). The full score distribution is illustrated in Figure 1, which shows zero inflation at score 0 and the near absence of participants at score 1 (*n* = 4; 0.4%).

Classical item analysis provides evidence for the construct validity and unidimensionality of the Knowledge Total composite. Item difficulty (proportion endorsing each hazard) ranged from 0.075 (K4, aquaculture residues) to 0.284 (K1, general health risk), indicating all items are difficult—consistent with the low overall awareness observed. Corrected item-total correlations ranged from 0.498 (K4) to 0.853 (K1), all substantially exceeding the recommended threshold of 0.30, indicating adequate discrimination for all items. Alpha-if-item-deleted values ranged from 0.804 to 0.868, with no item substantially improving internal consistency upon deletion (Cronbach’s *α* = 0.865 for the full scale). Principal component analysis (PCA) on the binary item matrix—serving as a classical proxy for exploratory factor analysis— yielded a dominant first factor explaining 64.8% of total variance, with a first-to-second eigenvalue ratio of 6.15 (threshold for strong unidimensionality: >5; 77). All six items loaded positively on Factor 1 (loadings: 0.204–0.596). Bartlett’s test of sphericity confirmed that the inter-item correlation matrix was factorable (χ^2^[15] = 2,980.28, *p* < 0.001). Collectively, these results support a single dominant factor structure and justify the use of the composite Knowledge Total score. Full Item Response Theory (IRT) modeling is beyond the scope of the present cross-sectional KAP survey, but is recommended for future scale development.

### 3.5 Predictors of high-risk consumption (Model 1)

The logistic regression model was statistically significant (χ^2^(8) = 219.927, *p* < 0.001) with Nagelkerke R^2^ = 0.259, AUC = 0.760, and satisfactory calibration (H–L: χ^2^(8) = 12.154, *p* = 0.144; all VIF ≤ 1.53). Final model results are presented in Table 8.

**Table 8:** Multivariable logistic regression: predictors of high-risk seafood consumption (Model 1; N = 1,021).

Predictor variable	*p*-value	OR	95% CI
Coastal residence (Yes vs. No)	<0.001	3.019	2.265–4.024
Age group (ref: 20–29 years)	<0.001	—	—
30–39 years	<0.001	2.302	1.583–3.347
40–49 years	<0.001	3.188	2.115–4.805
≥50 years	<0.001	3.494	2.014–6.062
Knowledge Total score	0.950	0.997	0.917–1.084
Educational level (ref: <bachelor’s)	<0.001	—	—
Bachelor’s degree	<0.001	0.539	0.384–0.757
Postgraduate	<0.001	0.385	0.244–0.606
Gender (female vs. male)	<0.001	0.534	0.395–0.723

OR, odds ratio; CI, confidence interval. Reference categories: non-coastal; 20–29 years; below bachelor’s degree. High-risk consumer = any consumption of *S. commerson* OR weekly-or-more consumption of *E. coioides*, *P. pessuliferus*, or *L. nebulosus* (Section 2.5.1). Model fit: χ^2^(8) = 219.927, *p* < 0.001; Nagelkerke R^2^ = 0.259; AUC = 0.760; H–L χ^2^(8) = 12.154, *p* = 0.144. VIF range 1.07–1.53.

Coastal residence was the strongest single predictor (OR = 3.019, 95% CI [2.265–4.024], *p* < 0.001). A clear monotonic age gradient was observed: 30–39 years (OR = 2.302, 95% CI [1.583–3.347]); 40–49 years (OR = 3.188, 95% CI [2.115–4.805]); ≥50 years (OR = 3.494, 95% CI [2.014–6.062]); all *p* < 0.001. Educational attainment showed a protective dose–response pattern: bachelor’s degree (OR = 0.539, 95% CI [0.384–0.757]) and postgraduate qualifications (OR = 0.385, 95% CI [0.244–0.606]), both *p* < 0.001. Objective food-safety knowledge was not significantly associated with high-risk consumption (OR = 0.997, 95% CI [0.917–1.084], *p* = 0.950). Sex was an independent predictor of high-risk consumption: women had significantly lower odds than men (OR = 0.534, 95% CI [0.395–0.723], p < 0.001), after full adjustment for all covariates. Sensitivity analyses across five alternative high-risk operationalizations (prevalence range: 24.9–70.4%) consistently reproduced the knowledge null association (OR range: 0.939–1.023; all *p* > 0.05; Supplementary Table S2).

Sex was tested and included in the final model. Female vs. Male OR = 0.534 (95% CI [0.395–0.723], *p* < 0.001); women had significantly lower odds of high-risk consumption than men after full adjustment for coastal residence, age, educational level, and knowledge score.

### 3.6 Predictors of mercury risk awareness (Model 2)

**Figure 2:**
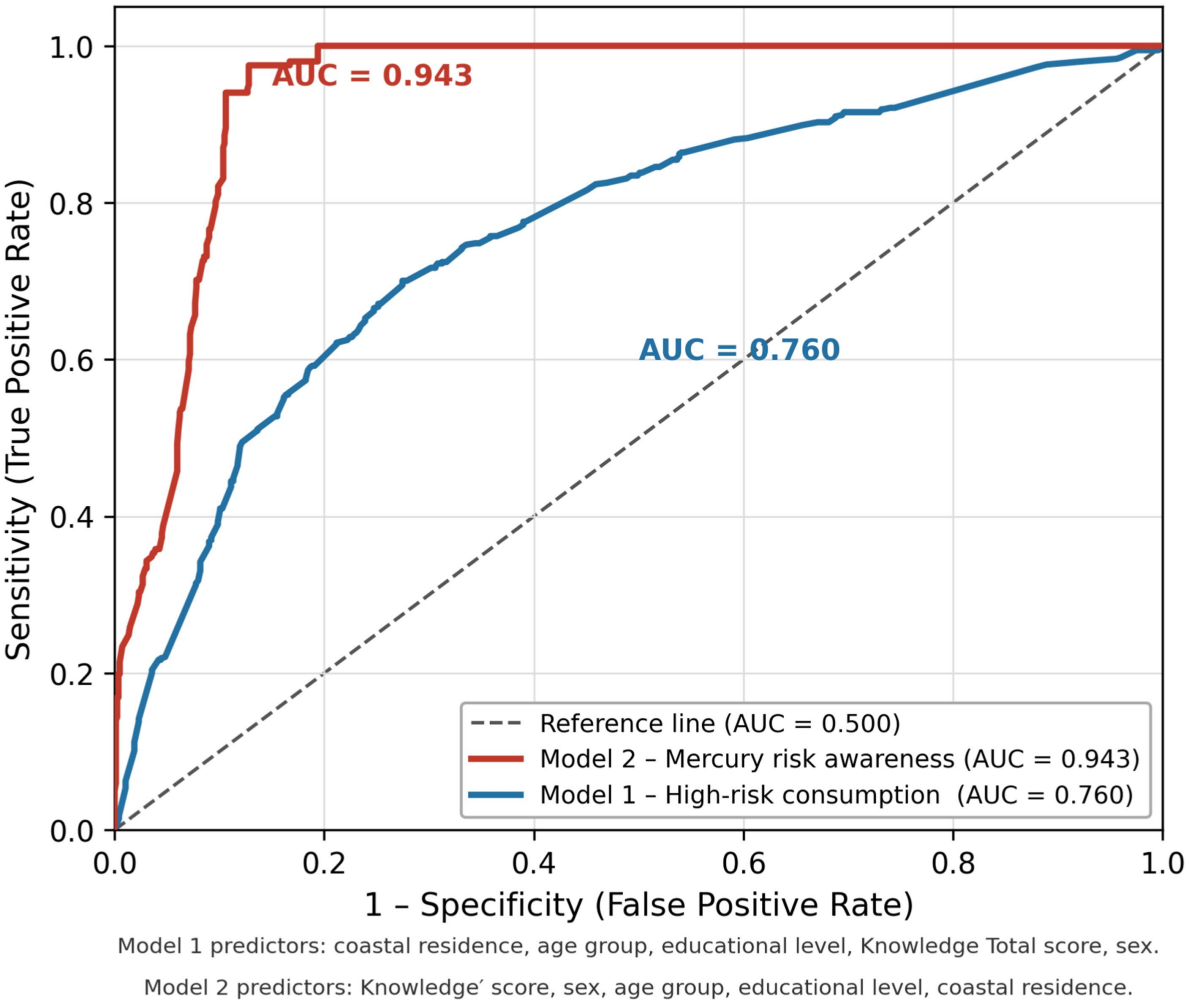
Receiver-operating characteristic (ROC) curves for Model 1 (high-risk seafood consumption; AUC = 0.760) and Model 2 (mercury risk awareness; AUC = 0.943). The substantially higher AUC in Model 2 reflects a ceiling effect driven by the zero-inflated Knowledge′ distribution (71.6% scoring zero), not by demographic covariates, which contributed only +0.009 in AUC (Knowledge′-only model: AUC = 0.934; full model: AUC = 0.943). Model 1 predictors: coastal residence, age group, educational level, Knowledge Total score, and sex. Model 2 predictors: Knowledge′ score, sex, age group, educational level, and coastal residence.

The mercury risk awareness model used Knowledge′ (*α* = 0.836; mean = 0.78 ± 1.38; 71.6% scoring zero) as the knowledge predictor to avoid circular dependency with the K2 outcome. The model achieved excellent discrimination (AUC = 0.943; χ^2^(8) = 462.92, *p* < 0.001; Nagelkerke R^2^ = 0.579). The exceptionally high AUC reflects the severely zero-inflated distribution of Knowledge′: participants scoring zero were almost uniformly mercury-unaware, while even a single unit increment in Knowledge′ was associated with near-fourfold higher odds of mercury awareness (OR = 4.000). A sensitivity analysis using a Knowledge′-only model confirmed that demographic covariates contributed minimally to overall discrimination (Knowledge′-only AUC = 0.934; full model AUC = 0.943; marginal gain = +0.009). The Spiegelhalter Z-statistic (Z = 1.60, *p* = 0.109) confirmed satisfactory calibration; the H–L χ^2^(8) = 122.80, *p* < 0.001 is attributable to well-documented over-rejection in large samples with highly skewed predictors (66). All VIF ≤ 1.61. Results are presented in Table 9. The ROC curves for both models are presented in Figure 2.

**Table 9:** Multivariable logistic regression: predictors of mercury risk awareness (Model 2; *N* = 1,021).

Predictor variable	*p*-value	OR	95% CI
Knowledge′ score (K1, K3–K6)	<0.001	4.000	3.350–4.777
Sex (female vs. male)	0.108	0.657	0.393–1.097
Age group (ref: 20–29 years)	0.583	—	—
30–39 years	0.334	1.364	0.727–2.559
40–49 years	0.734	1.128	0.564–2.258
≥50 years	0.982	1.010	0.407–2.507
Educational level (ref: <bachelor’s)	0.189	—	—
Bachelor’s degree	0.599	0.862	0.495–1.501
Postgraduate	0.056	1.960	0.982–3.911
Coastal residence (Yes vs. No)	0.274	0.769	0.480–1.231

Knowledge′ = K1 + K3 + K4 + K5 + K6 (K2 excluded to avoid circular dependency); range 0–5; α = 0.836. OR = 4.000 is the exact regression output (exp[1.386] = 4.000); retained as reported. Model fit: χ^2^(8) = 462.92, *p* < 0.001; Nagelkerke R^2^ = 0.579; AUC = 0.943. H–L χ^2^(8) = 122.80, *p* < 0.001 (large-sample over-rejection; Spiegelhalter Z = 1.60, *p* = 0.109 confirms adequate calibration (67)). VIF range 1.03–1.61.

After adjusting for Knowledge′, sex was not independently associated with mercury awareness (OR = 0.657, 95% CI [0.393–1.097], *p* = 0.108), indicating that the bivariate sex advantage was explained by women’s higher general food-safety knowledge rather than any sex-specific predisposition toward toxicological concern. Age, educational attainment, and coastal residence were not independently associated with mercury awareness (all *p* > 0.05).

## 4 Discussion

### 4.1 Overview of principal findings

The present investigation provides the first population-level characterization of the seafood-related MeHg knowledge–behavior gap among Saudi adults. The central empirical finding is the marked dissociation between the two regression models: food-safety knowledge strongly predicted mercury risk awareness (OR = 4.000 per unit) yet was entirely unassociated with high-risk species selection (OR = 0.997, *p* = 0.950). This dissociation was robust across five alternative operationalizations of the high-risk outcome (prevalence range 24.9–70.4%), confirming that it is structural rather than artefactual in character, and consistent with the broader knowledge–behavior gap literature (35, 43, 44). The ROC curves comparing the discriminative ability of the two models are shown in Figure 2.

### 4.2 Normalization of seafood consumption and the secondary role of safety

The finding that health and safety concerns were cited as barriers to consumption by only 7.0% of respondents illustrates how far toxicological concerns sit from the actual moment of purchase. This configuration—frequent consumption alongside minimal safety concern—closely mirrors observations from an Italian coastal population (38) and from US studies documenting limited advisory uptake (32, 33). Because seafood poses no ambient risk to this population, advisory messages about mercury hazards lack the salience needed to engage the decision processes that govern species selection (35). This interpretation is theoretically coherent with behavior-change frameworks that position food choice at the intersection of attitudes, subjective norms, perceived behavioral control, and practical feasibility—not information alone—a configuration consistent with the Theory of Planned Behavior (TPB), which posits that dietary intentions are driven by attitudes, subjective norms, and perceived behavioral control rather than information availability (37, 68).

The absence of widespread health-related avoidance also carries an important policy implication: seafood risk communication in Saudi Arabia is not occurring against a backdrop of public alarm or indiscriminate avoidance. This reduces the risk of beneficial seafood intake being inadvertently suppressed by risk messaging—an outcome documented among US pregnant women following national advisory releases (69)—but equally indicates that current advisory content lacks sufficient salience to modify species selection at the population level.

### 4.3 Species profile and exposure-relevant consumption

The prevalence of *E. coioides* (Al-Hamour; 44.9%) and *S. commerson* (Al-Kanaad; 44.6%) in participants’ reported diets is epidemiologically meaningful. Both species occupy upper trophic levels in Gulf food webs; monitoring studies consistently document mercury concentrations that exceed those of lower-trophic alternatives (49–52, 55, 70). These behavioral patterns are consistent with elevated exposure relevance when placed alongside hair mercury biomonitoring data from Gulf coastal communities—Okati and Esmaili-Sari (71) documented geometric mean hair mercury concentrations of 2.1–3.8 μg/g in Persian Gulf residents where predatory species consumption was the primary determinant of individual burden, and Al-Saleh et al. (59–61) report concentrations in Saudi women that exceed the WHO provisional tolerable weekly intake threshold in a substantial subset (71, 72) and risk assessments from Qatar and Kuwait, which demonstrate that species selection substantially determines individual-level MeHg exposure in the region (56–58).

The nuanced interpretation of tuna consumption—which varies markedly in mercury burden by species and product form, with Gulf canned light tuna averaging 0.09–0.28 ppm (64)—reinforces the need for product-sensitive communication rather than categorical advisories by food group. Atlantic salmon and European seabass, reported by 32.6 and 9.7% of participants, respectively, are primarily farmed or imported species generally low in mercury in their commercial form (7), supporting their exclusion from the high-risk classification. The present study did not collect biomarker data, so individual-level toxicological conclusions cannot be drawn; biomarker-based follow-up research in this population is a priority.

### 4.4 A robust knowledge–behavior gap

The robustness of the knowledge–behavior null association across five alternative operationalizations substantially strengthens the conclusion: the finding is not an artifact of how “high-risk” was defined. Information exerts no detectable influence on species selection at the point of purchase. This replicates findings from North American (31, 32), European (36), and Asian contexts (39), and is theoretically consistent with Ser and Watanabe’s “communication paradox” (43): toxicity messaging may raise abstract concern without displacing established food habits. Behavioral barriers—habit, cost, and preparation inconvenience—substantially outweigh safety concerns and reflect the dominant role of practical food-choice determinants (44–46).

This pattern aligns with the broader literature on contaminant communication. Jacobs et al. (73) demonstrated that trust in regulatory food safety systems reduces consumers’ perceived need to appraise contaminant risks. Burger and Gochfeld (31) established that formal educational attainment is not a reliable proxy for practical fish-safety literacy—a finding echoed here: while education independently protected against high-risk consumption, this effect operated through general health literacy rather than through MeHg-specific knowledge.

### 4.5 Coastal residence as a structural predictor

After adjusting for all covariates, coastal residence more than tripled the odds of high-risk consumption (OR = 3.019)—the largest effect in Model 1. This likely reflects greater proximity to local fish markets (the primary purchase point for 75.6% of the sample), familiarity with locally harvested predatory species, and the embedding of seafood within coastal dietary routines and culturally meaningful food practices (42). Gulf-region biomonitoring confirms that this geographic patterning has direct exposure implications: coastal consumers demonstrate higher hair mercury concentrations than inland counterparts (58, 71). High-risk consumption in this context should not be framed as an individual information failure; it is, rather, a structural outcome of the food environment and cultural food practices.

### 4.6 Age gradient and protective role of education

Age produced the most striking dose–response relationship in the model, with adjusted odds of high-risk consumption climbing from OR = 2.302 (30–39 years) to OR = 3.494among those aged ≥50 years—a nearly fourfold elevation relative to the youngest reference group. This pattern likely reflects decades of established dietary habit, cohort-level attachment to familiar Gulf species, and lower engagement with contemporary digital health communication (42). Educational attainment showed a consistent protective pattern: odds of high-risk consumption were 46% lower among bachelor’s degree holders and 62% lower among postgraduate-educated participants. This protective effect likely operates through general health literacy and information-seeking behavior rather than through MeHg-specific knowledge, given that education was not independently associated with mercury risk awareness after adjustment (32, 37).

### 4.7 Sex differences in consumption behavior and mercury awareness

Women’s higher unadjusted mercury awareness (23.6% vs. 16.5%) aligns with a cross-national pattern in which women report stronger engagement with household food-safety information, possibly reflecting caregiving roles and primary food-purchasing responsibility (33, 74). Notably, the sex effect operated differently across the two outcome models. In Model 1, female sex was an independent protective predictor of high-risk consumption after full adjustment (OR = 0.534, 95% CI [0.395–0.723], *p* < 0.001), indicating that women were substantially less likely to engage in high-risk species consumption even after controlling for coastal residence, age, education, and knowledge. This behavioral protection did not translate into a knowledge advantage in Model 2: once food-safety knowledge was included, the sex difference in mercury awareness disappeared (OR = 0.657, *p* = 0.108), suggesting that women’s lower high-risk consumption reflects behavioral or cultural food-choice patterns rather than superior toxicological knowledge. Women were more mercury-aware because they knew more about food safety in general, not because sex independently confers toxicological concern. This is consequential for program design: interventions investing in broad food-safety literacy are likely to be more effective across both sexes than those targeting women as a demographic category alone (36, 44).

### 4.8 Implications for public health communication and policy

Three strategic priorities emerge. First, communication must shift from abstract toxicological education to species-specific, actionable guidance. Messages should identify—by local Arabic market name—the species to limit (Kanaad) and those that exceed advisory frequency thresholds (Hamour, Najil, Shaour), alongside lower-mercury alternatives widely available in Saudi markets (shrimp, salmon, seabass, rabbitfish, tilapia). Lando and Zhang (32) demonstrated that species-level—rather than general mercury hazard—knowledge is the cognitive threshold required for behavior change, a finding directly applicable to the Saudi context.

Geographic focus is the second priority. Because coastal residence drives high-risk consumption more powerfully than any cognitive variable (OR = 3.019), the most efficient use of public health resources is to place species-specific advisories where coastal consumers actually buy fish: in the traditional fish markets that serve as primary purchase points for 75.6% of the sample. Jeddah, Dammam, Jizan, Jubail, and Yanbu represent natural anchor cities for such a program, consistent with evidence on effective food environment interventions (37, 43).

Third, integrating seafood risk guidance into maternal health services and primary care would extend targeted reach to older adults and those with lower educational attainment. Integration into antenatal care is particularly important given documented neurodevelopmental vulnerability associated with prenatal MeHg exposure (13, 23), a consideration that has shaped advisory design in comparable contexts (69, 75). The Saudi Ministry of Health’s existing dietary guidance channels (48) provide an established infrastructure for disseminating species-level content to priority groups.

### 4.9 Comparison with international literature

The present findings extend a consistent global pattern. Like the Italian coastal study (38), we document frequent consumption of predatory species alongside limited toxicological awareness. Like US advisory uptake studies (32, 33), we find that partial awareness does not translate into safer dietary choices. As in European multicountry assessments (34), we observe variation in risk awareness attributable to knowledge rather than to demographic factors per se. The Arab–Gulf region adds distinctive elements: a dietary tradition centered on predatory species with particularly high regional mercury concentrations (51, 55), a rapidly expanding seafood supply chain under Vision 2030 (3, 47), and a population in which urban-digital connectivity may co-exist with deeply established traditional food practices. These specificities underscore the need for regionally adapted—rather than globally transferred—advisory and communication strategies.

### 4.10 Limitations and strengths

Four limitations warrant acknowledgment. First, convenience sampling through social-media channels yielded a sample with 74.5% university graduates, far exceeding the Saudi national average (~22–25%) (76); this almost certainly inflates mercury awareness estimates (19.7% is likely an overestimate) and deflates high-risk consumption rates (53.3% is likely an underestimate), making our findings conservative lower bounds for the structural gap. Second, open-channel distribution precluded response-rate calculation, and participant nationality was not collected; given that expatriates constitute approximately 38% of Saudi residents (76), inference is limited primarily to Saudi nationals. Third, mercury risk awareness was captured by a single yes/no item, adequate for hazard recognition but insufficient for multi-dimensional risk perception; social desirability bias and question-ordering effects cannot be excluded. Fourth, the absence of biomarker data means exposure is characterized behaviorally rather than confirmed biochemically; additionally, we did not collect data on current pregnancy status, precluding specific sub-group analysis of this highly vulnerable demographic; data collection overlapped with Ramadan 2026 (approximately 1–30 March 2026), introducing a potential seasonal bias unlikely to alter the direction of the knowledge–behavior gap; and the cross-sectional design precludes causal inference.

Several methodological strengths support the validity of this investigation. This is the first study to survey adults across all 13 Saudi administrative regions simultaneously. The final sample (n = 1,021) substantially exceeded the minimum requirement and yielded EPV values well above conventional thresholds. The high-risk consumption variable was operationalized using species-specific FDA/U.S. EPA advisory thresholds corroborated by Gulf-region contamination data, and its robustness was confirmed across five alternative specifications. The dual-model analytical design enabled direct detection of the knowledge–behavior dissociation. Non-parametric confirmation of parametric findings and calibration assessment via the Spiegelhalter Z-statistic (67) provided additional methodological rigor.

## 5 Conclusion

Across 1,021 adults spanning all 13 Saudi administrative regions, this nationwide investigation has documented a structural knowledge–behavior gap in seafood-related MeHg exposure. Over half the respondents met species-based criteria for high-risk consumption; fewer than one in five recognized mercury as a health concern; and the food-safety knowledge that reliably predicted awareness had essentially no influence on dietary choice. Coastal residence, advancing age, female sex, and lower educational attainment independently predicted high-risk consumption—none of which are cognitive or informational in character. The knowledge–behavior null association was robust across five alternative operationalizations (sensitivity range: 24.9–70.4%; all knowledge ORs non-significant), confirming the gap is structurally determined.

The central public health challenge is not a lack of available fish-safety information but the structural and habitual barriers that prevent existing information from influencing the food environments and practices through which species selection actually occurs. Addressing this challenge requires species-named advisories embedded in coastal fish retail environments, clinical integration targeting antenatal and older adult populations, and sustained investment in population health literacy. Future research incorporating biomarker-based exposure measurement, probability sampling, validated multi-item KAP instruments, and longitudinal designs would substantially strengthen causal inference and provide a firmer evidentiary foundation for targeted interventions in Saudi Arabia and the broader Gulf region.

## Data Availability

The original contributions presented in the study are included in the article/Supplementary material, further inquiries can be directed to the corresponding author.
