# Supplementary material for: Seafood consumption patterns and methylmercury risk awareness among Saudi adults: a nationwide cross-sectional survey documenting a structural knowledge–behavior gap
Source: Front Public Health. 2026 Jul 20;14:1886816. doi: 10.3389/fpubh.2026.1886816 (PMC13429608; doi:10.3389/fpubh.2026.1886816)
Supplement: Supplementary file 1 [file Data_Sheet_1.docx]

**Supplementary File S3**

**Survey Instrument (English Version)**

**Study:** Seafood Consumption Patterns and Methylmercury Risk Awareness among Saudi Adults: A Nationwide Cross-Sectional Survey Documenting a Structural Knowledge–Behavior Gap

**Journal:** Frontiers in Public Health | Environmental Health and Exposome

**Authors:** Nawaf W. Alruwaili, Abdulaziz Mashraqi, Nora Alafif

**Ethics:** King Saud University (Ref. KSU-HE-26-0045)

**Funding:** Ongoing Research Funding Program (ORF-2026-1554), King Saud University

────────────────────────────────────────────────────────────────

**Instrument Notes**

1. Administered electronically via Google Forms, January–April 2026.

2. Deployed bilingually (Arabic/English). This file presents the English source text used as the basis for the four-stage forward–backward translation described in Section 2.4 of the main manuscript.

3. Adapted from Spagnolo et al. (2025; Environments 12:66) with species substitutions and risk-awareness items adjusted to the Saudi context.

4. Items K1–K6 (Q18) constitute the Food-Safety Knowledge composite. K2 (marked ★) is the binary outcome for Model 2 in the main manuscript and was excluded from Knowledge′ to prevent circular dependency.

5. Pilot test with 30 adults (excluded from analysis) confirmed item clarity before full deployment.

6. No personally identifiable data was collected. Survey completion implied consent.

────────────────────────────────────────────────────────────────

# Participant Preamble (displayed at survey start)

**Dear participant,**

You are invited to participate in a research study examining seafood consumption patterns and awareness of health risks associated with fish consumption among adults in Saudi Arabia. This study is conducted by the Department of Community Health Sciences, College of Applied Medical Sciences, King Saud University, and has received full ethical approval (Ref. KSU-HE-26-0045).

Participation is entirely voluntary and anonymous. You may withdraw at any point without consequence. No personally identifiable information is collected. Completing and submitting this form constitutes your informed consent to participate. The survey takes approximately 8–10 minutes to complete.

For enquiries: nalruwaili@ksu.edu.sa

────────────────────────────────────────────────────────────────

# Section 1: Sociodemographic Characteristics

**Q1. What is your sex?**

☐ Male

☐ Female

**Q2. What is your age group?**

☐ 20–29 years

☐ 30–39 years

☐ 40–49 years

☐ 50–59 years

☐ 60 years or older

**Q3. What is your marital status?**

☐ Single

☐ Married

☐ Divorced

☐ Widowed

**Q4. In which Saudi administrative region do you currently reside?**

☐ Riyadh

☐ Makkah (including Jeddah)

☐ Al-Madinah

☐ Al-Qassim

☐ Eastern Province

☐ Asir

☐ Tabuk

☐ Ha'il

☐ Northern Borders

☐ Jazan

☐ Najran

☐ Al-Baha

☐ Al-Jawf

**Q5. Do you reside in a coastal or inland city?**

*Coastal city = a city situated on the Red Sea or Arabian Gulf littoral.*

☐ Coastal city

☐ Inland city

**Q6. What is the highest level of education you have completed?**

☐ Below secondary school

☐ Secondary school / high school

☐ Diploma

☐ Bachelor's degree

☐ Higher diploma (postgraduate)

☐ Master's degree

☐ Doctoral degree (PhD)

**Q7. How many people live in your household (including yourself)?**

☐ 1–3 members

☐ 4–6 members

☐ 7 or more members

**Q8. Are there any children under 18 years of age living in your household?**

☐ Yes

☐ No

**Q9. Are you the primary person responsible for grocery shopping in your household?**

☐ Yes

☐ No

# Section 2: Seafood Consumption Behavior

**Q10. Do you consider yourself a regular seafood consumer?**

☐ Yes

☐ No

**Q11. How often do you typically consume each of the following types of seafood?**

*Please select one response per row.*

|  | **Never** | **Rarely (<monthly)** | **Monthly** | **Bi-weekly** | **Weekly** | **Several times/week** | **Daily** |
| --- | --- | --- | --- | --- | --- | --- | --- |
| Fresh seafood | ☐ | ☐ | ☐ | ☐ | ☐ | ☐ | ☐ |
| Frozen seafood | ☐ | ☐ | ☐ | ☐ | ☐ | ☐ | ☐ |
| Canned seafood (e.g., canned tuna) | ☐ | ☐ | ☐ | ☐ | ☐ | ☐ | ☐ |

**Q12. What is your typical portion size of seafood per eating occasion?**

*One standard serving ≈ 100–150 g (approximately the size of a deck of cards).*

☐ ≤100 g

☐ 101–125 g

☐ 126–150 g

☐ 151–175 g

☐ 176–212.5 g

☐ ≥250 g

**Q13. Where do you usually purchase seafood?**

*(Select all that apply)*

☐ Local fish market

☐ Supermarket or hypermarket

☐ Discount store

☐ Online platform

☐ Other: ___________________

**Q14. What primarily drives your seafood purchasing decisions?**

*(Select all that apply)*

☐ Quality and freshness

☐ Price / affordability

☐ Health considerations and origin

☐ Ease of preparation

☐ Availability / convenience

**Q15. What are the main reasons you do not consume more seafood than you currently do?**

*(Select all that apply)*

☐ Lack of dietary habit or routine

☐ High price

☐ Preparation challenges

☐ Taste preference

☐ Health and safety concerns

☐ Limited availability

☐ Other: ___________________

**Q16. Which of the following fish and seafood types do you usually consume?**

*† Species with documented elevated mercury concentrations in Arabian Gulf/Red Sea waters based on published environmental monitoring studies.*

*(Select all that apply)*

☐ Tuna (Thunnus spp.)

☐ Shrimp (Penaeus spp.)

☐ Greasy grouper — Al-Hamour (Epinephelus coioides) †

☐ Narrow-barred Spanish mackerel — Al-Kanaad (Scomberomorus commerson) †

☐ Atlantic salmon (Salmo salar)

☐ Spangled emperor — Al-Shaour (Lethrinus nebulosus) †

☐ Leopard coral grouper — Al-Najil (Plectropomus pessuliferus) †

☐ European seabass (Dicentrarchus labrax)

☐ White-spotted spinefoot — Sijan (Siganus canaliculatus)

☐ Other: ___________________

# Section 3: Food-Safety Knowledge and Methylmercury Risk Awareness

**Q17. Do you believe that consuming seafood carries any health risks?**

☐ Yes

☐ No

☐ Unsure

**Q18. Which of the following health hazards do you associate with seafood consumption?**

*Reviewer note: Items K1–K6 constitute the Food-Safety Knowledge composite (Knowledge Total, range 0–6; Cronbach’s α = 0.865). K2 (★) is the binary mercury risk awareness outcome for Model 2 in the main manuscript and was excluded from Knowledge′ (K1+K3+K4+K5+K6) to prevent circular dependency with the outcome variable.*

*(Select all that apply)*

☐ K1 General health risk from seafood (e.g., allergic reactions, food poisoning)

☐ K2 ★ Mercury or heavy metals [← MODEL 2 OUTCOME ITEM]

☐ K3 Microorganisms (bacteria, viruses, parasites)

☐ K4 Veterinary drugs or aquaculture residues

☐ K5 Preservatives or storage compounds

☐ K6 Raw or undercooked fish

☐ None of the above — I do not associate seafood with any health hazard

**Q19. Are you aware of any government or health authority recommendations regarding the type or frequency of fish consumption for health or safety reasons?**

☐ Yes

☐ No

☐ Unsure

**Q20. If YES to Q19: from which source did you primarily receive that information?**

*Answer only if you responded Yes to Q19.*

☐ Saudi Ministry of Health

☐ Healthcare provider or doctor

☐ Television or radio

☐ Internet or social media

☐ Family or friends

☐ Other: ___________________

**Q21. Have you ever changed your seafood consumption habits (species, frequency, or portion size) as a result of health or safety information?**

☐ Yes — I eat less seafood overall

☐ Yes — I avoid specific species

☐ Yes — I reduced portion size or frequency

☐ No — health information has not changed my seafood habits

☐ Unsure

────────────────────────────────────────────────────────────────

**End of questionnaire. Thank you for your participation.**

nalruwaili@ksu.edu.sa | King Saud University | ORF-2026-1554

────────────────────────────────────────────────────────────────

# Appendix: Variable-to-Question Mapping

Maps each survey question to the dataset variable name and its analytical role in the main manuscript.

| **Question** | **Item Description** | **Dataset Variable** | **Role in Analysis** |
| --- | --- | --- | --- |
| Q1 | Sex | Gender | Candidate predictor — Models 1 & 2 |
| Q2 | Age group | Age4 (4 levels) | Predictor — Models 1 & 2 |
| Q3 | Marital status | Marital_Status | Descriptive only |
| Q4 | Region | Region (1–13) | Descriptive; Table S8 |
| Q5 | Coastal/inland | Coastal_City | Predictor — Models 1 & 2 |
| Q6 | Educational level | Edu3 (3 levels) | Predictor — Models 1 & 2 |
| Q7 | Household size | Household_Size | Descriptive only |
| Q8 | Children present | Children_Present | Descriptive only |
| Q9 | Primary shopper | Primary_Shopper | Descriptive only |
| Q10 | Regular consumer | Regular_Consumption | Descriptive; Section 3.2 |
| Q11 | Consumption frequency | Freq_Fresh/Frozen/Canned | Table 2; weekly frequency composites |
| Q12 | Portion size | Portion_Size | Table 3 |
| Q13 | Purchase location | Purchase_* flags | Table 4 |
| Q14 | Purchasing drivers | Factor_* flags | Section 3.2 |
| Q15 | Barriers to consumption | Reason_* flags | Table 5 |
| Q16 | Species consumed | Fish_* binary flags | Table 6; high-risk outcome operationalization |
| Q17 | General risk belief | Health_Risk_Belief | Descriptive |
| Q18 — K1 | General health risk | K1_Risk_Aware | Knowledge Total; Knowledge′ |
| Q18 — K2 ★ | Mercury/heavy metals | **K2_Mercury** | Outcome: Model 2. Excluded from Knowledge′. |
| Q18 — K3 | Microorganisms | K3_Microorganisms | Knowledge Total; Knowledge′ |
| Q18 — K4 | Veterinary drugs | K4_Antibiotics | Knowledge Total; Knowledge′ |
| Q18 — K5 | Preservatives | K5_Preservatives | Knowledge Total; Knowledge′ |
| Q18 — K6 | Raw/undercooked fish | K6_Raw_Fish | Knowledge Total; Knowledge′ |
| Q19–Q21 | Advisory awareness & behavior change | Perception_Score composite | Descriptive |

Knowledge Total = K1+K2+K3+K4+K5+K6 (range 0–6; α = 0.865). Knowledge′ = K1+K3+K4+K5+K6 (range 0–5; α = 0.836). High-risk outcome = any S. commerson consumption OR weekly-or-more E. coioides / P. pessuliferus / L. nebulosus consumption (Section 2.5.1). ★ K2 is the binary mercury awareness outcome (Model 2 only).
