# Supplementary material for: Seafood consumption patterns and methylmercury risk awareness among Saudi adults: a nationwide cross-sectional survey documenting a structural knowledge–behavior gap
Source: Front Public Health. 2026 Jul 20;14:1886816. doi: 10.3389/fpubh.2026.1886816 (PMC13429608; doi:10.3389/fpubh.2026.1886816)
Supplement: Supplementary file 2 [file Table_1.pdf]

Supplementary Table S1

STROBE Statement — Checklist of Items That Should Be Included in Reports of Cross-Sectional Studies

Seafood Consumption Patterns and Methylmercury Risk Awareness among Saudi Adults: A Nationwide Cross-Sectional Survey Documenting a Structural Knowledge–Behavior Gap

Alruwaili NW, Mashraqi A, Alafif N | Frontiers in Public Health | Environmental Health and Exposome | ORF-2026-1554

**Section/Topic**	**Item No.**	**Recommendation**	**Reported in Manuscript**
Title and abstract	1(a)	Indicate the study design with a commonly used term in the title or the abstract	Title: "…A Nationwide Cross-Sectional Survey…"
	1(b)	Provide an informative and balanced summary of what was done and what was found	Abstract (lines 16–40; 268 words): structured with explicit inline headings Background, Methods, Results, Conclusion per reviewer request; conclusion sentence explicitly ties to stated study objective
Introduction			
Background/rationale	2	Explain the scientific background and rationale for the investigation being reported	Section 1 (Introduction): nutritional benefits of seafood; MeHg toxicology and neurodevelopmental risk; advisory frameworks; knowledge–behavior gap; Saudi Arabia context
Objectives	3	State specific objectives, including any pre-specified hypotheses	Section 1, final paragraph. Hypothesis explicitly stated: "We hypothesized that objective food-safety knowledge would be positively associated with both higher mercury risk awareness and lower high-risk seafood consumption after adjustment for relevant sociodemographic covariates." Three study objectives enumerated.
Methods			
Study design	4	Present key elements of study design early in the paper	Section 2.1: "A descriptive cross-sectional survey design was adopted."
Setting	5	Describe the setting, locations, and relevant dates, including periods of recruitment, exposure, follow-up, and data collection	Section 2.2: Kingdom of Saudi Arabia, all 13 administrative regions; coastal and inland areas; data collection January–April 2026
Participants	6	Give the eligibility criteria, and the sources and methods of selection of participants	Section 2.2 (lines 118–140): adults ≥20 years residing in Saudi Arabia; age threshold of 20 years justified by: independent food-purchasing status, alignment with source instrument [38] and prior Saudi dietary KAP surveys, and institutional ethics requirements (KSU-HE-26-0045); bilingual

Section/Topic	Item No.	Recommendation	Reported in Manuscript
			(Arabic/English) Google Forms; social-media distribution; participation explicitly stated as self-selected/voluntary opt-in; regional coverage across all 13 regions noted as non-population-proportional
Variables	7	Clearly define all outcomes, exposures, predictors, potential confounders, and effect modifiers; give diagnostic criteria, if applicable	Section 2.5.1: high-risk outcome (species-specific FDA/U.S. EPA thresholds)<br><br>Section 2.5.2: Knowledge Total and Knowledge′ composites (K1–K6)<br><br>Section 2.5.3: mercury risk awareness (K2 binary item)<br><br>Section 2.6: predictors — age, sex, education, coastal residence, knowledge
Data sources / measurement	8	For each variable of interest, give sources of data and details of methods of assessment; describe comparability of assessment methods if more than one group	Section 2.4: instrument adapted from Spagnolo et al. (2025); four-stage forward–backward translation; pilot test with n=30<br><br>Section 2.5: all variables operationalized from survey items<br><br>File S3 (supplementary): full bilingual survey instrument
Bias	9	Describe any efforts to address potential sources of bias	Section 2.2 (lines 118–140): factual sampling procedure only; interpretive bias language consolidated in Limitations<br>Section 2.6 (lines 207–232): sex included in Model 1; OR = 0.534 (95% CI [0.395–0.723], p < 0.001) reported in Table 8 footnote (lines 362–365)<br>Section 4.10 Limitations and Strengths (lines 564–591): retitled; limitations precede strengths; four consolidated limitations:<br>Limitation 1 (≈lines 565–569): convenience/education sampling bias — 74.5% graduates vs ~22–25% national average; awareness 19.7% is overestimate; high-risk 53.3% is underestimate; gap is conservative lower bound<br>Limitation 2 (≈lines 570–572): sampling constraints — response-rate calculation precluded; ~38% of Saudi residents are expatriates [64]; inference limited to Saudi nationals<br>Limitation 3 (≈lines 573–574): measurement limitations — single yes/no awareness item; social desirability bias and sensitization cannot be excluded; any sensitization would narrow (not widen) the gap<br>Limitation 4 (≈lines 575–581): design limitations — no biomarker data; Ramadan 2026 overlap (1–30 March); cross-sectional design precludes causal inference<br><br>Section 2.6: sex was tested as a candidate predictor and **retained** as an independent predictor in the final Model 1 (Enter/forced-entry method, 8 predictors, all retained). Adjusted estimate reported in Table 8 footnote: Female vs. Male OR = 0.534 (95% CI [0.395–0.723], p < 0.001); women had significantly lower odds of high-risk consumption than men after full adjustment for coastal residence, age, educational level, and knowledge score (Lines 362–365)<br><br>Section 4.10 (Limitations and Strengths — limitations precede strengths; consolidated to 4): Limitation 1 — convenience/education sampling bias (overestimates awareness, underestimates high-risk prevalence); Limitation 2

Section/Topic	Item No.	Recommendation	Reported in Manuscript
			— response-rate and expatriate/nationality constraints; Limitation 3 — single-item measurement and possible response bias; Limitation 4 — absence of biomarker data, pregnancy-status data, Ramadan 2026 overlap, and cross-sectional design (Lines 565–581)
Study size	10	Explain how the study size was arrived at	Section 2.3: Cochran's formula (Z=1.96; P=0.50; d=0.05; 95% CI) → minimum n=384; target raised to n=425 for non-response; achieved n=1,021. Post-hoc EPV: **68 (Model 1, 8 predictors)**; 25 (Model 2, 8 predictors); both exceed the recommended minimum of 10.
Quantitative variables	11	Explain how quantitative variables were handled in the analyses; if applicable, describe which groupings were chosen and why	Section 2.5: knowledge items scored dichotomously (0/1) and summed into Knowledge Total (0–6) and Knowledge′ (0–5)<br><br>Section 2.6: age grouped into 4 levels (20–29, 30–39, 40–49, ≥50); education grouped into 3 levels (<bachelor's, bachelor's, postgraduate); both groupings justified by sample distribution and advisory relevance
Statistical methods	12(a)	Describe all statistical methods, including those used to control for confounding	Section 2.6 (lines 207–232): Pearson chi-square; independent-samples t-tests; Mann–Whitney U (non-parametric verification); two binary logistic regression models estimated using Enter (forced-entry) method; Nagelkerke R^2; AUC; Hosmer–Lemeshow test; Spiegelhalter Z-statistic [76]; VIF multicollinearity check. IBM SPSS Statistics Version 30.0 [66]
	12(b)	Describe any methods used to examine subgroups and interactions	Section 2.6 (lines 207–232): sex included as predictor in both Model 1 and Model 2; all predictors retained in final models<br>Section 3.3 (lines 279–286): sex-stratified bivariate analyses (Table 7)<br><br>Section 3.3: sex-stratified bivariate analysis (chi-square, t-test, Mann–Whitney U)
	12(c)	Explain how missing data were addressed	No missing data in any analytical variable. Complete case analysis on N=1,021. Incomplete submissions operationally defined in Section 2.2 (≈lines 133–136, within S2.2 paragraph lines 118–140): sessions in which one or more mandatory items were not answered before session termination; Google Forms prevented submission unless all required fields were completed — all 1,021 retained responses are complete on all mandatory items
	12(d)	If applicable, describe analytical methods taking into account the sampling strategy	Section 2.2: non-probability, self-selected convenience sampling explicitly acknowledged; directional implications for each prevalence estimate discussed in Section 4.10
	12(e)	Describe any sensitivity analyses	Sections 2.6 and 3.5: five alternative operationalizations of the primary high-risk outcome (prevalence range 24.9%–70.4%); Knowledge Total OR non-significant in all five specifications (OR range 0.939–1.023; all p>0.05). Detailed results in Table S2.
Results			

Section/Topic	Item No.	Recommendation	Reported in Manuscript
Participants	13(a)	Report numbers of individuals at each stage of study	Section 2.2 (lines 118–140): ~1,078 responses initiated; ~57 excluded by Google Forms automatic incomplete-submission filter; final analytic sample n = 1,021. Formal response-rate calculation precluded by open-channel social-media distribution
	13(b)	Give reasons for non-participation at each stage	Section 2.2 (lines 133–136): exclusion criterion formally defined — incomplete submissions = form sessions where one or more mandatory fields were not answered before session termination; Google Forms automatically prevented submission of any form with unanswered required items, so no partial record was ever exported to the dataset. ~57 excluded by this platform-level filter. Open-channel distribution precluded formal response-rate calculation and systematic non-response bias assessment. Acknowledged in Section 4.10 Limitation 2
	13(c)	Consider the use of a flow diagram	Not applicable — open social-media survey with no multi-stage eligibility screening requiring participant-flow tracking
Descriptive data	14(a)	Give characteristics of study participants and information on exposures and potential confounders	Table 1 (sociodemographic characteristics); Section 3.1; Tables 2–6 (consumption patterns, species, purchasing)
	14(b)	Indicate the number of participants with missing data for each variable of interest	No missing data in any analytical variable (confirmed for all N=1,021 cases)
Outcome data	15	Report the number of outcome events or summary measures	Section 3.3: high-risk consumption n=544 (53.3%); mercury awareness n=201 (19.7%)<br><br>Sections 3.2–3.6; Tables 7–9
Main results	16(a)	Give unadjusted estimates and, if applicable, confounder-adjusted estimates and their precision; make clear which confounders were adjusted for and why they were included	Tables 8–9 (lines 355–393): adjusted ORs with 95% CIs for all predictors. Model 1 final: $\chi^2(8) = 219.927$, $p < 0.001$; Nagelkerke $R^2 = 0.259$; AUC = 0.760; sex retained (Female OR = 0.534, $p < 0.001$; Table 8 footnote lines 362–365). Model 2: $\chi^2(8) = 462.92$, $p < 0.001$; Nagelkerke $R^2 = 0.579$; AUC = 0.943. Confounders: age, sex, educational level, coastal residence; all retained in final models<br><br>Adjusted: Tables 8–9 (main manuscript); full coefficients including unstandardized B, SE, and Wald χ^2 in Tables S5–S6 (Supplementary)<br><br>Sections 3.5–3.6 (Lines 362–365, 426–428, 456–457)<br><br>Model 1: $\chi^2(8)$=219.927, p<0.001; Nagelkerke R^2=0.259; AUC=0.760; H–L $\chi^2(8)$=12.154, p=0.144 (Table 8; Lines 362–365)<br><br>Sex adjusted OR (Table 8 footnote, Lines 362–365): Female vs. Male OR = **0.534** (95% CI [0.395–0.723], p < 0.001); women had significantly lower odds of high-risk consumption than men after full adjustment for coastal residence, age, educational level, and knowledge score

Section/Topic	Item No.	Recommendation	Reported in Manuscript
			Confounders/predictors retained in Model 1: coastal residence, age, sex, educational level, Knowledge Total; included on theoretical grounds and prior literature
	16(b)	Report category boundaries when continuous variables were categorized	Section 2.5: age groups (20–29, 30–39, 40–49, ≥50 years; reference: 20–29); education (below bachelor's, bachelor's, postgraduate; reference: below bachelor's)<br><br>Table 8 footnote: reference categories stated explicitly (non-coastal; 20–29 years; below bachelor's degree)
	16(c)	If relevant, consider translating estimates of relative risk into absolute risk for a meaningful time period	Not applicable — cross-sectional design; relative risk cannot be estimated; no incidence data
Other analyses	17	Report other analyses done — e.g., analyses of subgroups and interactions, and sensitivity analyses	Section 3.4 (lines 293–322): Knowledge Total score distribution as standalone subsection, including:<br>(a) Zero-inflation: 71.6% (n=731) scored zero; mean = 0.97 ± 1.68; median = 0 (lines 294–304)<br>(b) Psychometric analysis paragraph (R3 revision; lines 305–322):<br>• Item difficulty: K1=0.284 to K4=0.075<br>• Corrected item-total r: 0.498–0.853 (all > 0.30)<br>• Alpha-if-item-deleted: 0.804–0.868<br>• PCA: Factor 1 = 64.8% variance; F1/F2 ratio = 6.15 (>5 = strongly unidimensional); all items load positively on Factor 1<br>• Bartlett's test: $\chi^2(15) = 2{,}980.28$, $p < 0.001$<br>• IRT noted as future direction<br>Figure 1: Knowledge Total histogram (lines 332–337)<br>Figure 2: ROC curves (lines 406–413)<br>Table S2: Sensitivity analysis across five alternative outcome operationalizations<br><br>Figure 1: Histogram of Knowledge Total score distribution (N=1,021) — displays zero-inflation (71.6% at score 0) and near-absence at score 1 (0.4%; n=4) (Line 312)<br><br>Figure 2: ROC curves for Model 1 (AUC=**0.760**) and Model 2 (AUC=0.943) (Lines 406–413)<br><br>Section 3.3: sex-stratified bivariate analyses<br><br>Section 3.5: five-definition sensitivity analysis; Knowledge Total OR range 0.939–1.023 across all definitions<br><br>Table S2: full sensitivity analysis results<br><br>Tables S3–S6: full bivariate and regression coefficient tables
Discussion			

Section/Topic	Item No.	Recommendation	Reported in Manuscript
Key results	18	Summarize key results with reference to study objectives	Section 4.1: overview of principal findings; two-model dissociation; structural determination of gap
Limitations	19	Discuss limitations of the study, taking into account sources of potential bias or imprecision; discuss both direction and magnitude of any potential bias	Section 4.10 Limitations and Strengths — retitled; limitations precede strengths; lines 564–591:<br>Limitation 1 (≈lines 565–569): convenience/education sampling bias — 74.5% graduates vs ~22–25% national average [64]; awareness 19.7% is overestimate; high-risk 53.3% is underestimate; gap is conservative lower bound<br>Limitation 2 (≈lines 570–572): sampling constraints — response-rate calculation precluded; ~38% of Saudi residents are expatriates [64]; inference limited primarily to Saudi nationals<br>Limitation 3 (≈lines 573–574): measurement limitations — single yes/no awareness item; social desirability bias and sensitization effects cannot be excluded<br>Limitation 4 (≈lines 575–581): design limitations — no biomarker data (plausible not confirmed exposure); Ramadan 2026 overlap (approximately 1–30 March 2026); cross-sectional design precludes causal inference<br><br>Limitation 1 (Lines 645–686): Convenience/education sampling bias — 74.5% university graduates vs. ~22–25% national average [64]; awareness 19.7% is an overestimate; high-risk 53.3% is an underestimate; gap is a conservative lower bound<br><br>Limitation 2: Sampling constraints — formal response-rate calculation precluded; nationality (Saudi vs. non-Saudi expatriate) not collected; ~38% of Saudi residents are expatriates [64], and fish-consumption habits differ across cultural backgrounds (e.g., East Asian populations); inference limited primarily to Saudi nationals<br><br>Limitation 3 (Lines 573–574): Measurement limitations — mercury awareness captured by single yes/no item (adequate for hazard recognition; insufficient for multi-dimensional risk perception); social desirability bias and sensitization effects cannot be excluded, though any sensitization would narrow the gap (conservative effect)<br><br>Limitation 4 (Lines 575–581): Design limitations — no biomarker data (plausible, not confirmed, exposure); pregnancy status not collected, precluding sub-group analysis of this vulnerable demographic; Ramadan 2026 overlap (approximately 1–30 March 2026; unlikely to alter gap direction); cross-sectional design precludes causal inference
Interpretation	20	Give a cautious overall interpretation of results considering objectives, limitations, multiplicity of analyses, results from similar studies, and other relevant evidence	Sections 4.1–4.10: findings contextualized against Italian, US, European, and Asian literature; structural vs. informational framing; three policy priorities identified
Generalizability	21	Discuss the generalizability (external validity) of the study results	Section 2.2 (lines 118–140): educated, urban-weighted sample; findings informative for hypothesis generation but not population-representative<br>Section 4.10 (lines 564–591): external validity caveats elaborated; future probability-sample studies recommended

Section/Topic	Item No.	Recommendation	Reported in Manuscript
			Section 5 Conclusion (lines 593–612): external validity caveats reiterated; future biomarker-based and longitudinal designs recommended<br><br>Section 4.10: external validity caveats elaborated; future probability-sample studies recommended<br><br>Section 5 (Conclusion): external validity caveats reiterated
Other information			
Funding	22	Give the source of funding and the role of the funders for the present study and, if applicable, for the original study on which the present article is based	Funding section: Ongoing Research Funding Program (ORF-2026-1554), King Saud University, Riyadh, Saudi Arabia. The funders had no role in study design, data collection or analysis, data interpretation, manuscript writing, or the decision to publish.

STROBE = Strengthening the Reporting of Observational Studies in Epidemiology. This checklist should be read in conjunction with the STROBE explanation and elaboration document (Vandenbroucke JP et al. Ann Intern Med. 2007;147:W163–W194). EPV = events per variable. VIF = variance inflation factor. AUC = area under the receiver-operating characteristic curve. “Generalizability” uses American English per Frontiers in Public Health style guidelines.
