# Supplementary material for: Seafood consumption patterns and methylmercury risk awareness among Saudi adults: a nationwide cross-sectional survey documenting a structural knowledge–behavior gap
Source: Front Public Health. 2026 Jul 20;14:1886816. doi: 10.3389/fpubh.2026.1886816 (PMC13429608; doi:10.3389/fpubh.2026.1886816)
Supplement: Supplementary file 3 [file Table_2.docx]

**Supplementary Table S2**

**Sensitivity Analysis: Knowledge Total Score as a Predictor of High-Risk Seafood Consumption Across Five Alternative Outcome Operationalizations**

Seafood Consumption Patterns and Methylmercury Risk Awareness among Saudi Adults: A Nationwide Cross-Sectional Survey Documenting a Structural Knowledge–Behavior Gap

*Alruwaili NW, Mashraqi A, Alafif N | Frontiers in Public Health | Environmental Health and Exposome | ORF-2026-1554*

**Rationale:**

The primary high-risk outcome (Definition 1) classifies a participant as a high-risk consumer if they reported (a) any consumption of Scomberomorus commerson (Al-Kanaad; designated "Choices to Avoid" by FDA/U.S. EPA, mean mercury ≥0.46 ppm) OR (b) weekly-or-more consumption of Epinephelus coioides (Al-Hamour), Plectropomus pessuliferus (Al-Najil), or Lethrinus nebulosus (Al-Shaour) — all "Good Choices" with a recommended ceiling of one serving per week. To assess whether the null association between objective food-safety knowledge and high-risk consumption was an artifact of this operationalization, four alternative definitions were pre-specified and tested. All five models include the same covariate set as Model 1 of the main manuscript: coastal residence, age group (4 levels; reference: 20–29 years), educational level (3 levels; reference: below bachelor's degree), and Knowledge Total score. Sex was retained in all five models as a covariate, consistent with the final Model 1 specification in the main manuscript. For Definition 1 (Primary), female sex was an independent predictor of high-risk consumption (OR = 0.534, 95% CI [0.395–0.723], p < 0.001). Sex ORs for Definitions 2–5 are not reported individually as the sensitivity analysis focuses on Knowledge Total; full coefficients are available upon request. Referenced in Sections 2.6 and 3.5 of the main manuscript.

| **Outcome Definition** | **Prevalence n (%)** | **Knowledge Total OR** | **95% CI** | **p-Value** | **Coastal Residence OR** | **Age ≥50 Years OR** | **Bachelor's Degree OR** | **Nagelkerke R² / AUC** |
| --- | --- | --- | --- | --- | --- | --- | --- | --- |
| **Definition 1: Definition 1 — Primary (main manuscript)** Any consumption of Al-Kanaad (S. commerson) [FDA/U.S. EPA "Choices to Avoid"; Gulf Hg: 0.45–1.20 ppm] OR weekly-or-more consumption of Al-Hamour (E. coioides), Al-Najil (P. pessuliferus), or Al-Shaour (L. nebulosus) [FDA/U.S. EPA "Good Choices"; ≤1 serving/week advised] | 53.3% (n = 544) | 0.997 | 0.917–1.084 | 0.950 | 3.019 | 3.494 | 0.539 | 0.259 / 0.760 |
| **Definition 2: Definition 2 — Strictest (Kanaad only)** Any consumption of Al-Kanaad (S. commerson) only, regardless of frequency. Tests whether the null finding holds when restricted to the single species most clearly designated "Choices to Avoid" under FDA/U.S. EPA guidance. | 44.0% (n = 449) | **0.950** | **0.874–1.033** | **0.227** | 2.057 | 2.877 | 0.560 | 0.168 / 0.710 |
| **Definition 3: Definition 3 — Broadest (any predatory species)** Any consumption of Al-Kanaad OR any consumption of Al-Hamour, Al-Najil, or Al-Shaour, regardless of frequency. Tests the null finding under the broadest possible species-based classification, removing the frequency threshold for "Good Choice" species. | 70.4% (n = 719) | **0.939** | **0.865–1.019** | **0.132** | 3.000 | 3.808 | 0.687 | 0.181 / 0.728 |
| **Definition 4: Definition 4 — Tuna included** Definition 1 criteria PLUS weekly-or-more consumption of canned tuna (Thunnus spp.). Tests whether adding tuna (excluded from the primary definition due to variable Hg burden by product form) alters the knowledge–consumption association. | 69.5% (n = 710) | **1.015** | **0.935–1.101** | **0.727** | 2.180 | 2.587 | 0.563 | 0.106 / 0.669 |
| **Definition 5: Definition 5 — Frequency only; Al-Kanaad excluded** Weekly-or-more consumption of Al-Hamour, Al-Najil, or Al-Shaour only; Al-Kanaad excluded. Tests the knowledge–consumption null finding using a purely frequency-based criterion without the "any consumption" threshold applied to Al-Kanaad in the primary definition. | 24.9% (n = 254) | **1.023** | **0.925–1.131** | **0.664** | 4.516 | 3.474 | 0.636 | 0.203 / 0.745 |
| Summary across all 5 definitions | Range: 24.9%–70.4% | Range: 0.939–1.023 | **All include null value (1.0)** | **All p > 0.05** | Range: 2.057–3.019 All p < 0.001 | Range: 2.587–3.808 All p < 0.001 | Range: 0.539–0.687 All p < 0.001 | — |

**OR,** Odds ratio from binary logistic regression.

**CI,** 95% confidence interval.

**AUC,** Area under the receiver-operating characteristic curve.

**Nagelkerke R²,** Pseudo-R² for the full covariate model.

**All models,** Adjusted for coastal residence, age group (reference: 20–29 years), educational level (reference: below bachelor's degree), and Knowledge Total score. Sex (female vs. male) retained in all five models per the final Model 1 specification; OR = 0.534 (95% CI [0.395–0.723], p < 0.001) for Definition 1. Full sex coefficients in main Table 8.

**Knowledge Total,** Sum of K1+K2+K3+K4+K5+K6 (range 0–6; Cronbach's α = 0.865). Cronbach's α is equivalent to Kuder–Richardson Formula 20 for binary items.

**Coastal Residence OR,** Odds ratio for coastal vs. inland city residence. Reference: inland city.

**Age ≥50 OR,** Odds ratio for the ≥50-year age group. Reference: 20–29 years. Note: full age gradient reported in main Table 8.

**Bachelor's Degree OR,** Odds ratio for bachelor's degree vs. below bachelor's. Postgraduate OR reported in main Table 8.

**Key finding,** Knowledge Total OR was non-significant in all five outcome definitions (OR range: 0.939–1.023; all 95% CIs span 1.0; all p > 0.05). In contrast, coastal residence, older age, and lower educational attainment were consistently significant across all five specifications (all p < 0.001). The structural knowledge–behavior gap documented in the main manuscript is not an artifact of the primary outcome operationalization.

**Species,** Al-Kanaad = Scomberomorus commerson; Al-Hamour = Epinephelus coioides; Al-Najil = Plectropomus pessuliferus; Al-Shaour = Lethrinus nebulosus.

**Hg,** Mercury. ppm = parts per million (mg/kg wet weight).
